# *Drosophila melanogaster* as a High-Throughput Model for Host–Microbiota Interactions

**DOI:** 10.3389/fmicb.2017.00751

**Published:** 2017-04-28

**Authors:** Mark Trinder, Brendan A. Daisley, Josh S. Dube, Gregor Reid

**Affiliations:** ^1^Centre for Human Microbiome and Probiotic Research, Lawson Health Research Institute, St. Joseph’s Health Care London, LondonON, Canada; ^2^Schulich School of Medicine and Dentistry, Department of Microbiology and Immunology, University of Western Ontario, LondonON, Canada; ^3^Schulich School of Medicine and Dentistry, Department of Surgery, University of Western Ontario, LondonON, Canada

**Keywords:** microbiota, microbiome, *Drosophila melanogaster*, animal model, fruit fly, probiotic, prebiotic, symbiosis

## Abstract

Microbiota research often assumes that differences in abundance and identity of microorganisms have unique influences on host physiology. To test this concept mechanistically, germ-free mice are colonized with microbial communities to assess causation. Due to the cost, infrastructure challenges, and time-consuming nature of germ-free mouse models, an alternative approach is needed to investigate host–microbial interactions. *Drosophila melanogaster* (fruit flies) can be used as a high throughput *in vivo* screening model of host–microbiome interactions as they are affordable, convenient, and replicable. *D. melanogaster* were essential in discovering components of the innate immune response to pathogens. However, axenic *D. melanogaster* can easily be generated for microbiome studies without the need for ethical considerations. The simplified microbiota structure enables researchers to evaluate permutations of how each microbial species within the microbiota contribute to host phenotypes of interest. This enables the possibility of thorough strain-level analysis of host and microbial properties relevant to physiological outcomes. Moreover, a wide range of mutant *D. melanogaster* strains can be affordably obtained from public stock centers. Given this, *D. melanogaster* can be used to identify candidate mechanisms of host–microbe symbioses relevant to pathogen exclusion, innate immunity modulation, diet, xenobiotics, and probiotic/prebiotic properties in a high throughput manner. This perspective comments on the most promising areas of microbiota research that could immediately benefit from using the *D. melanogaster* model.

## *Drosophila melanogaster* Model for Microbiota Studies

Next generation sequencing has increased the popularity and understanding of microbiota (community of microorganisms residing on/in a multicellular organism) contribution to host function in health and disease. Several diseases have been partially attributed to changes in microbiome composition such as *Clostridium difficile* associated diarrhea, diabetes mellitus, mood disorders, atherosclerosis, and others (Bäckhed et al., 2012). However, microbiota studies often assume differences in identity and abundance of certain host microorganisms promote and/or mitigate disease. This is shown by the growing number of reports on microbiota correlations that lack attempts to demonstrate causality.

A major challenge to experimental design is that the microbiota concept greatly increases the complexity of the reductionist approach in which a single pathogen is responsible for a given disease, as proven by Koch’s postulates (Byrd and Segre, 2016). The gold standard for demonstrating causality uses germ-free mice to demonstrate that a condition of interest is induced following host colonization with microbes associated with the aforementioned condition. However, major barriers to germ-free mouse studies are cost, lengthy study time, technical challenges, and infrastructure demands. These limitations also manifest in germ-free mouse studies often having low experimental sample sizes.

The field of probiotic and prebiotic science faces similar challenges. Probiotics are defined as “live microorganisms that, when administered in adequate amounts, confer a health benefit on the host” (Food and Agriculture Organization of the United Nations, and World Health Organization [FAO/WHO], 2002; Hill et al., 2014). High power clinical trials are required to draw reliable conclusions about probiotic efficacy. However, the cost and time required to perform clinical trials large enough to be informative have left many probiotic claims dependent *in vitro* studies. This results in scarce evidence as to whether some marketed probiotics are effective *in vivo.* This issue is further troubled by numerous microbes receiving generalized probiotic claims even though probiotic properties are often conferred in a strain-specific manner.

An alternative approach is needed to investigate microbiota and probiotic interactions in a living host. An ideal model organism would have the following characteristics: high throughput screening capabilities, inexpensive, fast reproduction, and an easily manipulated microbiome. *Drosophila melanogaster* stands out as an excellent model organism which possesses these qualities, and can allow reliable validation of probiotic effects in a living organism. Furthermore, many of the tools to investigate host–microbe relationships in *D. melanogaster* are already available due to its rich history in pathogen research (Lemaitre and Hoffmann, 2007).

**FIGURE 1:**
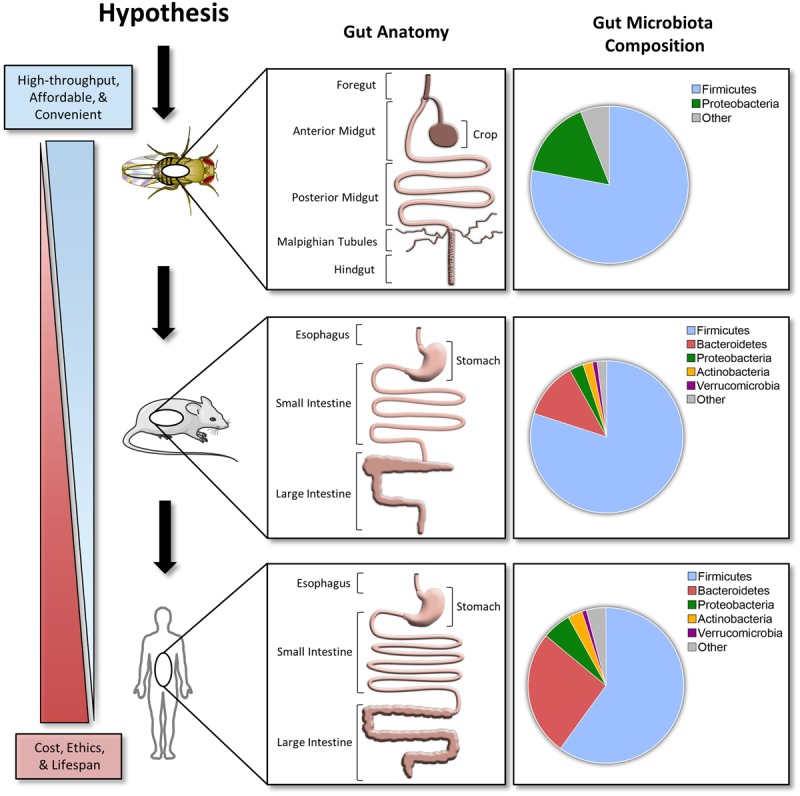
**A hypothesized pipeline approach investigating host–microbe interactions.** Comparisons between the *Drosophila melanogaster*, C57BL/6 mice, and human gut anatomy and microbiota. Taxonomical distribution data for top phyla in *D. melanogaster* are based off 454 tag pyrosequencing (Blum et al., 2013). C57BL/6 mice and human data are based off a 2.6 and 4.6 M catalog, respectively (Xiao et al., 2015). Stock clipart images from Servier Medical Art by Servier were used and modified under the Creative Commons Attribution 3.0 Unported License.

Compared to the mammalian gastrointestinal tract, the *D. melanogaster* gut has several major differences, but the overall structure and function are similar (**Figure 1**). The gastrointestinal physiology, anatomy, and signaling pathways controlling intestinal development, regeneration, and pathology are highly conserved in *D. melanogaster* (Apidianakis and Rahme, 2011). The details of *D. melanogaster* gastrointestinal tract are beyond the scope of this article, but can be found in the review cited (Lemaitre and Miguel-Aliaga, 2013).

The idea of using *D. melanogaster* for investigating symbiotic modulation of host physiology has been stated by others (Ryu et al., 2010; Storelli et al., 2011; Erkosar et al., 2013; Ma et al., 2015). However, use of the efficient and convenient *D. melanogaster* model for microbiota research has yet to be implemented widely. A wide range of *D. melanogaster* strains available in public repositories^1^ can be derived germ-free and maintained easily without the requirement of expensive animal facilities, equipment, and technicians (Koyle et al., 2016).

Compared to the mouse or human microbiota, the *D. melanogaster* microbiota is simple with a low microbial diversity (1–30 species) and is typically dominated by *Lactobacillus* and *Acetobacter* (Blum et al., 2013; Erkosar et al., 2013; Chaston et al., 2014). This simplified community structure deconvolutes the complexity of deciphering the effect of a given microbial species on the greater community and its host. Similar to the genomics era being initiated with assessment of small and simple genomes, microbiota research could benefit from a simplified model system such as *D. melanogaster* to deconstruct complex polymicrobial interactions *in vivo*. Taken together, *D. melanogaster* experimentation is affordable, convenient, and rarely requires approval by animal ethics review boards. These characteristics make *D. melanogaster* an ideal high-throughput *in vivo* model for understanding host–microbiota interactions.

## Microbiota-Mediated Pathogen Exclusion

**FIGURE 2:**
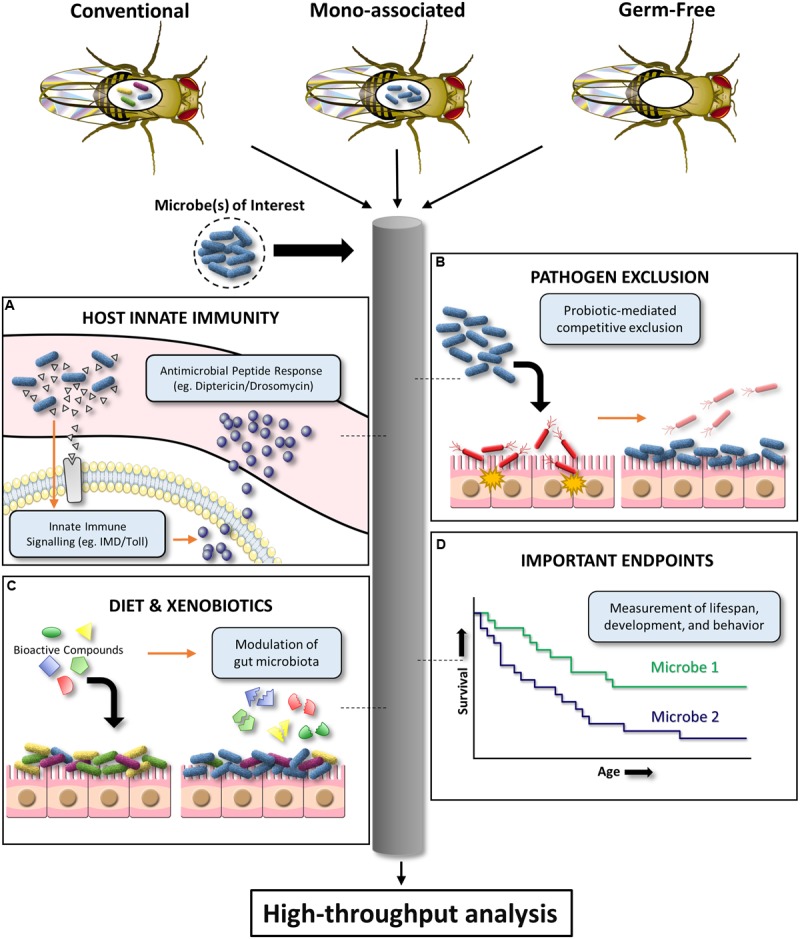
**Schematic diagram illustrating the high-throughput potential of *D. melanogaster* to investigate host–microbe interactions.** Conventional, mono-associated, and germ-free *D. melanogaster* can be used to mechanistically dissect the effects of a given microbe on **(A)** innate immune system signaling, **(B)** pathogen exclusion, **(C)** xenobiotic biotransformation and associated effects on the microbiota, and **(D)** important endpoints such as lifespan, development, and behavior.

Certain microbes can outcompete others via chemical inhibition, physical and nutritional competitive exclusion, and a variety of other adaptive mechanisms. In particular, the gut microbiota and probiotic organisms are critical for inhibiting intestinal microbial pathogenesis (Reid et al., 2011). However, promising *in vitro* screens to identify anti-pathogenic gut microbiota isolates are often unsuccessful upon further testing in expensive mammalian models. *D. melanogaster* has the potential to be an affordable, preliminary *in vivo* infection model for a variety of bacterial, fungal, and viral pathogens (Apidianakis and Rahme, 2009, 2011). The *D. melanogaster* infection model has been used to demonstrate pathogen inhibition by the human probiotic *L. rhamnosus* GG (Blum et al., 2013). This highlights the potential of *D. melanogaster* as a high throughput screening tool to substantiate *in vivo* pathogen inhibition claims for human probiotic or microbiota organisms of interest. Specifically, established oral or septic infection models can be used to assess the ability of different microbial communities to prevent pathogen-induced *D. melanogaster* colonization, persistence, and/or mortality (Neyen et al., 2014). This simplified model system also provides a powerful platform for elucidating ill-defined mechanisms of *in vivo* microbiota/probiotic-mediated pathogen inhibition (**Figure 2**).

Opportunistic infections are another field of study suitable for assessment in *D. melanogaster*. Gut microbiota commensals are known to become pathogenic under certain circumstances (Fei and Zhao, 2013; Cho et al., 2015). In commonly used experimental models, it is difficult to decipher the causative environmental, microbial, or host factors responsible for inducing this symbiotic-pathogenic shift. For example, the well-known intestinal opportunistic pathogen *Clostridium difficile* is triggered by an apparent reduction in microbial diversity induced by antibiotic treatment. Replacement of microbial diversity via fecal transplant has been a very effective strategy to overcome this disease, however, failure to identify a specific species or subset of species responsible for resolution of pathogenesis demands further investigation (van Nood et al., 2013). Using the available immunodeficient *D. melanogaster* stocks as an *in vivo* model (Neyen et al., 2014), researchers can better decipher the mechanistic triggers that determine how individual species transition from symbiotic-to-pathogenic.

## Microbiota Modulation of Host Innate Immunity

Despite lacking an adaptive immune system, *D. melanogaster* has been a crucial model for innate immunity discoveries. Findings of microbiota-mediated modulation of *D. melanogaster* local gut immunity (Ryu et al., 2010) provide evidence for using this model to evaluate microbial strain variations in innate immunoregulation. *D. melanogaster* immune responses to pathogen-associated molecular patterns can largely be separated into two distinct pathways: (i) The Toll pathway which is activated by lysine (Lys)-type peptidoglycan (Gram-positive bacteria) and β-1,3 glucan (fungi); (ii) The immune deficiency (IMD) pathway which is activated by diaminopimelic acid (DAP)-type (Gram-negative and Gram-positive bacilli bacteria) (Lemaitre and Hoffmann, 2007; Buchon et al., 2014). Together, the Toll and IMD pathways are critical for upregulating nuclear factor kappa-light-chain-enhancer of activated B cells (NF-κB) response genes, such as the antimicrobial peptides Drosomycin and Diptericin (**Figure 2**). Both *D. melanogaster* Diptericin and Drosomycin reporter strains are publicly available. Preliminary work has shown that these mutant flies can be used to screen microbes (single or in combination) for *in vivo* induction or repression of these conserved innate signaling pathways by measuring antimicrobial peptide readouts using a fluorescent microplate reader (for high-throughput interpretation) or microscope (for tissue localization inquiries). However, there are fundamental questions that require further clarification. The upstream transcription factor responsible for regulating Diptericin, Relish, has been shown to have pleiotropic roles in promoting host survival in response to noxious stimuli such as radiation (Karpac et al., 2011), and regulation of cell death (Chinchore et al., 2012). It is currently unknown how Relish modulation by the indigenous microbiota effects host physiology and what aspects of bacteria make this pathway targetable. The availability of IMD and Toll loss- and gain-of-function *D. melanogaster* mutants will be instrumental for addressing these questions (Neyen et al., 2014). Alternatively, multiplex quantitative RT-PCR can be used to affordably assess the effect of different microbial communities on the global RNA expression of a well-characterized *D. melanogaster* immunity gene panel (Neyen et al., 2014).

There are several other *D. melanogaster* assays, although less amendable to high throughput screening, that will be useful for investigating the interplay between microbial symbionts and host innate immunity. Melanin production occurs in the hemolymph (invertebrate equivalent to blood) of *D. melanogaster* to prevent microbial pathogenesis via encapsulation. Benoit et al. (2017) demonstrated that enteric symbionts of larval *D. melanogaster* regulated melanin production through activation of a highly conserved hematopoietic pathway. Though this study demonstrated yet another important role of the microbiota in host immunity, the specific microbes and signaling molecules responsible for these interactions have yet to be identified. Simple experiments testing one of the referenced melanization assays in mono-associated versus germ-free *D. melanogaster* could be used to address this line of inquiry (Neyen et al., 2014; Koyle et al., 2016). In addition, oxidative results in the production of reactive oxygen species (ROS) by *Duox* in the *D. melanogaster* gut and is crucial for immunity and microbiota regulation (Ryu et al., 2010). Abnormal commensalism appears to be detrimental to *D. melanogaster* due to consequential tissue damage associated with *Duox* overstimulation. The ability to assess ROS production by *D. melanogaster* in response to diverse microbial communities may provide valuable insights into how the microbiota regulates the balance of immune priming for pathogen eradication versus autoimmunity. Alternatively, evolutionary advancement toward more defensive *Wolbachia* (obligate endosymbionts) variants in *D. melanogaster* have been shown to increase resistance to *Drosophila* C virus (Faria et al., 2016). Thus, *D. melanogaster* could help to elucidate mechanisms by which symbiotic microbes affect the evolutionary trajectories of their host and improve scientific knowledge of cost-benefit relations associated with these mechanisms (Faria et al., 2015). In summary, *D. melanogaster* has potential to be used as a screening tool of microbe *in vivo* innate immunogenicity and as a model organism for evaluating distal site effects of crosstalk between the microbiota and innate immunity.

## Interplay of Microbiota with Diet and Xenobiotics

Diet strongly modulates the gut microbiota (David et al., 2014). However, current understanding of how this interaction affects host nutritional status and fitness is still fragmented. *D. melanogaster* can be used to study diet-microbiota-host interactions due to: (a) their simplistic gut microbiota, (b) consistent feeding behavior regardless of food composition (Partridge et al., 2005), and (c) the simple generation and maintenance of germ-free stocks (Koyle et al., 2016). Wong et al. (2014) took advantage of these favorable characteristics and demonstrated that the *D. melanogaster* gut microbiota spares dietary B-vitamins on low-yeast diets, promotes protein nutrition, and suppresses lipid storage on high sugar diets. Others have also utilized *D. melanogaster* to mechanistically assess the life-extending properties of caloric restriction (Lee et al., 2014) and reduction of intestinal bacterial loads during later life stages (Brummel et al., 2004). These studies elude to the crosstalk between diet and the host microbiota as a regulator of important physiological aspects of *D. melanogaster* physiology such as lifespan (Brummel et al., 2004) and development (Storelli et al., 2011). Future high-throughput studies combining minor modification to *D. melanogaster* nutritional components and detailed investigation of the host-microbe nutrient interactions hold promise for dietary science. High-throughput dietary assessment in *D. melanogaster* prior to verification of findings in rodents or humans enables researchers to identify the most promising compounds that would not be feasible in costlier and more time-consuming mouse or human studies. The high proportion *Lactobacillus* spp.—species of Gram-positive bacteria commonly used as probiotics—in the *D. melanogaster* gut microbiota makes this model promising for preliminary identification of novel prebiotics.

The gut microbiota can affect the biotransformation of many xenobiotics, but this has been largely overlooked by pharmacology and toxicology. Beyond the direct interaction with xenobiotics, the microbiota has been shown to modulate host xenobiotic metabolic processes (Spanogiannopoulos et al., 2016). Since cytochrome P450 enzymes (major drug metabolism family) vary between species (Sakai et al., 2015), *D. melanogaster* have rarely been used to assess human-targeted pharmaceuticals. Nevertheless, *D. melanogaster* offers a platform to screen for microbe-xenobiotic interactions *in vivo* (**Figure 2**). In addition, specific *in vivo* microbe–drug interactions can be tested by mono-associating germ-free *D. melanogaster* with the microbe of interest followed by exposure to drug(s). This system is particularly useful for assessing the interactions of microbes with environmental toxins or drug-induced toxicity, where lethality, growth impairment, or behavioral abnormalities can be used as high throughput readouts. For instance, sub-chronic pesticide exposure can cause off-target toxicity in humans and wildlife species such as honey bees (*Apis mellifera*). Microbes can be screened for the ability to mitigate or exacerbate the aforementioned signs of xenobiotic induced-toxicity using the approach exemplified with organophosphate pesticides (Trinder et al., 2016). Preliminary work in our lab using the neonicotinoid insecticide, imidacloprid, suggests *D. melanogaster* can also be used as a model to identify compounds that induce microbiota changes, and with the help of available mutant stocks also elucidate the mechanism of compound-induced microbiota modification. Therefore, despite obvious limitations to these types of experiments (e.g., transient nature of non-resident microbes in the *D. melanogaster* intestinal tract), using *D. melanogaster* to obtain preliminary data from high throughput screens of microbe–drug interactions could save time and resources, as well as provide data with more predictive value (Pandey and Nichols, 2011).

## Assessment of Probiotics and Prebiotics on Important Experimental Endpoints

The *D. melanogaster* microbiota is critical for modulating keystone properties of host health such as development (Storelli et al., 2011), lifespan (Brummel et al., 2004), and behavior (Sharon et al., 2010). These findings suggest that targetable microbiota modulation (e.g., probiotics, prebiotics, diet, antibiotics, etc.) may have predictive validity for understanding how microbiota alteration can influence aspects of human health and disease that are challenging to assess in mammals. In humans, *Lactobacillus* spp. and *Bifidobacterium* spp. are the most commonly used probiotic organisms capable of transiting through the harsh gastrointestinal tract to confer their host with physiological benefits. However, many probiotic organisms require further testing to determine strain-specific properties and mechanisms of action. Replication of *Lactobacillus plantarum*-induced *D. melanogaster* developmental growth resilience to malnutrition in mice provides evidence for this model’s insect-mammalian translation potential (Schwarzer et al., 2016). *D. melanogaster* provides a tool to evaluate the effect of extensive permutations of microbiota manipulation on important endpoints such as lifespan, development, and behavior in a cost-effective, timely, and feasible manner (Pandey and Nichols, 2011).

*Drosophila melanogaster* development progresses through well-defined stages of egg hatching, larval instar growth, pupae formation, and metamorphosis into an imago (adult fruit fly) in approximately 9 days. This rapid generation time enables classical recolonization studies of germ-free *D. melanogaster* to easily modify the dose and permutations of all microbiota constituents for assessment of their contribution to important host phenotypes singly or in combination. This feat is currently not possible with complex microbiotas such as those found in humans which can have 100–1000 different species of resident bacteria. Using this reductionist approach in *D. melanogaster* allows for easy identification of keystone species that correlate to host phenotypes of interest.

Prebiotic compounds could be conveniently added to *D. melanogaster* food media sources (most commonly cornmeal-molasses-yeast agar) and subsequently screened for induction of probiotic effects. In particular, mortality, body size, body weight, flight capability, stress, anesthesia response, activity, aggression, and fecundity are amendable to relatively high throughput screening in adult *D. melanogaster* (Pandey and Nichols, 2011). Alternatively, larval *D. melanogaster* can be used to assess mortality, body size, necrosis, and olfaction in a relatively high-throughput fashion (Pandey and Nichols, 2011). It is important to stress that work with *D. melanogaster* enables researchers to assess the most meaningful experimental endpoint, mortality, without the ethical conundrums of most other animal models. Furthermore, microbial species responsible for inducing host phenotypes of interest can be mechanistically followed-up using the large publicly available repositories (Bloomington *Drosophila* Stock Center, Kyoto Stock Center, etc.) of mutant *D. melanogaster* stocks.

## Future Directions and Conclusion

The affordable, high throughput, and experimental manipulability of *D. melanogaster* make it an ideal model for deciphering complex systems biology questions with a more reductionist approach, which would be unfeasible in other models (Supplementary Table 1). For instance, *D. melanogaster* will be an important tool for understanding how manipulation of multi-variable factors such as drugs, toxins, diet, and microbes effect the host and its associated microbiota. Established *D. melanogaster* models of human neurologic, cardiovascular, neoplastic, metabolic, and other diseases could potentially be used to study the reported contribution of the microbiota to these conditions. Specifically, the foundational history of *D. melanogaster* as a neurobiology model provides a wide variety of established methods for investigation of the gut-brain axis. Microbiota-mediated modulation of this axis has been linked to a wide range of neurological diseases including multiple sclerosis, depression, anxiety, and autism.

In addition, chronic human diseases may be partially attributable to early life microbiota acquisition epigenetic priming (Thorburn et al., 2015). The microbiota and its metabolites play a role in epigenetic modifications at critical time points during development that can have long lasting health effects. *D. melanogaster* appear to be an excellent model to efficiently assess how microbial inheritance could affect development, epigenetic modification, and disease heritability in successive generations. The proposed use of *D. melanogaster* as an *in vivo* model for drug discovery with greater predictive validity than *in vitro* assays suggests that this model could also be used to screen probiotic or prebiotic properties (Pandey and Nichols, 2011). However, experimental design and translation from the *D. melanogaster* microbiota model will require careful consideration due to major shortcomings of a simplified microbiota (Kostic et al., 2013), lack of adaptive immunity (Apidianakis and Rahme, 2011), and differences in gastrointestinal anatomy and physiology (Lemaitre and Miguel-Aliaga, 2013). In conclusion, *D. melanogaster* is an underutilized model for deciphering mechanisms of host–microbial symbiotic relationships. The simplified microbiota structure of *D. melanogaster* makes it amendable for developing tools, techniques, and knowledge required to advance this field.

## Author Contributions

MT, BD, JD, and GR conceived ideas for the manuscript. MT and BD drafted the manuscript. MT, BD, JD, and GR revised the manuscript. All authors agree to be accountable for the content of the work.

## Conflict of Interest Statement

The authors declare that the research was conducted in the absence of any commercial or financial relationships that could be construed as a potential conflict of interest.

## Footnotes

1 http://flybase.org/
