# Supplementary material for: Drosophila melanogaster as a High-Throughput Model for Host–Microbiota Interactions
Source: Front Microbiol. 2017 Apr 28;8:751. doi: 10.3389/fmicb.2017.00751 (PMC5408076; doi:10.3389/fmicb.2017.00751)
Supplement: Supplementary file 1 [file Table_1.PDF]

## SUPPLEMENTARY INFORMATION

**_Drosophila melanogaster_ as a high-throughput model for host-microbiota interactions.**

Mark Trinder#, Brendan A. Daisley#, Josh S. Dube, and Gregor Reid*.
# Denotes equal contribution.
* Correspondence: gregor@uwo.ca

**Supplementary Table 1.** Proposed uses of *D. melanogaster* as a discovery model for host-microbe interactions are highlighted. References provide experimental rationale for each section.

| | PROPOSED USE OF *D. MELANOGASTER* | REFERENCES |
|---|---|---|
| **PATHOGEN EXCLUSION** | • Use of established oral and septic infection models to assess ability of different microbial communities to prevent pathogen colonization, persistence, and/or mortality.<br>• Use of available knockouts to determine causal genetic factors implicated in host-mediated microbe association and pathogen susceptibility. | Apidianakis and Rahme, 2009<br>Apidianakis and Rahme, 2010<br>Blum et al., 2013 |
| **HOST INNATE IMMUNITY** | • Use of innate immune reporter fly lines (eg. flies expressing fluorescent diptericin/drosomycin) to evaluate microbe strain variations in innate immunoregulation via microplate (high-throughput interpretation) or microscope (for tissue localization inquiries) analyses.<br>• Use of the well-characterized *D. melanogaster* immunity gene panel to determine global RNA expression in response to different microbial communities. | Karpac et al., 2011<br>Neyen et al., 2014<br>Ryu et al., 2010<br>Sabat et al., 2015 |
| **DIET & XENOBIOTICS** | • Use of microbe-associated and germ-free *D. melanogaster* to quickly assess the impact of the microbiota on host dietary requirements and nutritional factors.<br>• Use of microbe-associated *D. melanogaster* to study the effects of a microbe(s) of interest on xenobiotic transformation and detoxification *in vivo*.<br>• Use of germ-free and mutant *D. melanogaster* to screen for microbe-mediated modulation of host detoxification. | Pandey and Nichols, 2011<br>Trinder et al., 2016<br>Wong et al., 2014 |
| **PROBIOTICS & PREBIOTICS** | • Use of probiotic-supplemented *D. melanogaster* to elucidate how microbes modulate keystone properties of host health such as development, lifespan, and behaviour.<br>• Use of prebiotic-supplemented *D. melanogaster* to investigate how non-digestible food ingredients differentially affect the growth of microbes *in vivo*.<br>• Use of microbe-associated and germ-free *D. melanogaster* to mechanistically determine how microbes affect a host phenotype of interest. | Brummel et al., 2004<br>Pandey and Nichols, 2011<br>Sharon et al., 2013<br>Storelli et al., 2011 |
